# Animated educational video using health belief model on the knowledge of anemia prevention among female adolescents: An intervention study

**DOI:** 10.51866/oa.136

**Published:** 2022-10-18

**Authors:** Siti Aisah, Suhartini Ismail, Ani Margawati

**Affiliations:** 1 S.Kp (Universitas Indonesia), MNS, PhD (Prince of Songkla University, Thailand) Department of Nursing, Emergency and Critical Care Nursing Division, Faculty of Medicine, Universitas Diponegoro, Jalan Prof. H. Soedarto, S.H. Tembalang, Semarang, Central Java, Indonesia. Email: suhartini.ismail@fk.undip.ac.id; 2 M. Kep., Sp. Kom (Universitas Indonesia), Doctor (Universitas Diponegoro, Indonesia), Doctoral Program of Medicine and Health Sciences, Faculty of Medicine, Universitas Diponegoro, Jalan Prof. H. Soedarto, S.H. Tembalang, Semarang, Central Java, Indonesia.; 3 Gerontology, Family and Community Nursing Department, Faculty of Nursing and Health Sciences Universitas Muhammadiyah Semarang, Jalan Kedungmundu Raya No.18 Semarang, Semarang, Central Java, Indonesia.; 4 Dra., M. Kes (Universitas Gajah Mada, Indonesia), PhD (The University of Hull, England), Department of Nutrition, Faculty of Medicine, Universitas Diponegoro Jalan Prof. H. Soedarto, S.H. Tembalang, Semarang, Central Java, Indonesia.

**Keywords:** Video-audio media, Anaemia, Prevention and control, Health belief model

## Abstract

**Introduction:**

As the younger female generation, female adolescents should understand anaemia prevention. This study examined the effects of animated educational videos on the knowledge of anaemia prevention among female adolescents using the Health Belief Model (HBM).

**Method:**

A quasi-experimental method with a randomised pre-test and post-test control group design was applied. Animated educational videos about anaemia prevention were used as the intervention. One hundred sixty-one female adolescents were recruited through multistage random sampling and divided into intervention (n=78) and control (n=83) groups. The intervention group received education via animated educational videos. The HBM questionnaire was used to measure the nine HBM indicators (r=0.8); the item categories were valid and reliable. Descriptive analyses, independent t-tests and repeated-measures ANOVA were used to analyse the data.

**Results:**

The animated educational videos played thrice significantly increased the knowledge of the intervention group (mean score: pre-test, 94; post-test one, 99; post-test two, 102). The scores for anaemia examination barriers (P=0.001), anaemia susceptibility (P=0.001), anaemia severity (P=0.001), anaemia prevention benefits (P=0.001), anaemia examination benefits (P=0.001), self-efficacy for obtaining iron tablets (P=0.001), self-recognition of anaemia signs and symptoms (P=0.001), signs of anaemia prevention (P=0.001) and health motivation (P=0.001) significantly changed. Meanwhile, the knowledge of the control group did not significantly increase (pre-test, 93; post-test one, 94; post-test two, 97). The intervention group had significantly higher mean scores in both the first and second measurements than the control group (P=0.05).

**Conclusion:**

Animated educational videos significantly increased the knowledge of anaemia prevention, including the nine HBM indicators.

## Introduction

Female adolescents are vulnerable to anaemia. According to the Indonesia Basic Health Research, the population with anaemia in Indonesia is dominated by patients aged 15–24 years, accounting for 32% of all populations in 2018.^1^ Classic physical examination of paleness can rule out and unassumingly rule in severe anaemia,^2^ allowing adolescents to indicate it by themselves. Female adolescents in Indonesia have a low level of knowledge on anaemia, including that of its definition, aetiology and prevention.^3^ Knowledge of anaemia is inversely proportional to the incidence of anaemia.

The mild impacts of anaemia include impaired growth, weakened cognitive and physical capacities, irregular menstrual cycle and decreased academic performance or low learning concentration.^4,5^ Meanwhile, severe impacts include decreased blood cell count, increased oxygen demand in cells, and impaired systemic blood circulation. Students who took iron supplements have been reported to have significantly increased math scores compared with those who did not. Thus, iron affects cognitive abilities and intelligence through increased concentration and attention.^6^

The Indonesia Ministry of Health, in collaboration with the United Nations Children’s Fund, has initiated weekly iron and folic acid supplementation, an anaemia management programme that focused on providing weekly iron tablets to female adolescents.^7^ However, this programme was not fully effective because of low interest in consumption of iron and folic acid supplementation, and the knowledge level about the function of iron tablets remains low.^8^ Increasing the knowledge of and motivating female adolescents to consume iron tablets regularly implemented to manage this problem.^9^ The Health Belief Model (HBM) is a theoretical model that can be used to guide health promotion and disease prevention programmes. Health education based on the HBM has been reported to significantly improve individual behaviours, habits and beliefs as well as knowledge, attitudes and practices.^10-12^

Previous findings evidenced that old media, such as leaflets, booklets and flipcharts, had less effectiveness to be used for the current generation. They highlighted that educational video media and simulation methods are more effective in increasing adolescent knowledge about anaemia than conventional media.^13^ Educational media using an animation video have been reported to effectively capture participants’ attention, increase their pleasure, contentment and knowledge and influence their behaviour owing to longer retention of information.^14, 15^

Therefore, the present study developed animated videos as informative, comprehensive and interesting health educational media to increase the knowledge of anaemia prevention among female adolescents.

## Methods

### Study design and participants

This study used a quantitative research method with a quasi-experimental randomised pretest and post-test design with a control group. It was performed in 2021 in Semarang City, Central Java, Indonesia. A four-stage random sampling method was used to select two senior high schools from a total of 12 senior high schools in Semarang City, with a 1.101 female student population. Two classes from two different schools were then selected randomly to recruit female students as the study’s specific sample. The sample size was calculated using the proportion formula developed by Lemeshow et al. (1990). The minimum sample size required for both intervention and control groups was 84, with an attrition consideration value of 20%.

A total of 161 female students were recruited from two senior high schools and assigned to intervention (n=78) and control (n=83) groups. The inclusion criteria were age of 14–19 years, ongoing menstrual cycle and absence of anaemia symptoms, such as anaemic conjunctiva and paleness on the face, lips, tongue, nails and palms. The signs and symptoms of anaemia were directly examined by conducting head-to-toe physical examination. The data were collected during task submission or COVID-19 protocol education in their schools. Paleness was identified as an abnormal skin colour on the face, lip, tongue, nails and palms.

### Video animation

The video contents were arranged on the basis of the qualitative data collection via interviews with 35 female adolescents using the following questions: ‘What is anaemia?’ and ‘What kind of video makes it easier for you to understand anaemia?’ The interview was conducted via multiple techniques (online and offline). Recorded interviews was analysed, and was used for formulating video content. All relevant information was first explained to the professional animation video creator to ensure that the video details were not missed.

A 10-minute video duration consist of general anaemia information, such as definition, pathophysiology, signs and symptoms and aetiology. The content was adapted from Becker’s (1974) HBM with nine indicators, which included the following variables: disease susceptibility, severity, health motivation and self-efficacy. Another part included specific information about anaemia, such as the impact, prevention and management or treatment of anaemia. The HBM was also applied to the material in part two on the following variables: perceived severity, benefits, barriers, cues to act, self-efficacy and health motivation. It included the substances at the primary, secondary and tertiary preventive levels.

### Data collection

The data were collected online using Gforms and the website system of education for female adolescent (web-SeaRP). Informed consent was obtained from the participants by sending a free Jotform application link. The intervention group was required to watch the video three times and undergo first post-test after they had finished watching and second post-test 4 weeks after the previous. Meanwhile, the control group received education using the video intervention after the research activities were completed.

The questionnaire consisted of items on anaemia prevention that were adapted and modified from a previous study.^16^ Pearson product-moment correlation coefficients were used to test the validity and reliability of the Indonesian version (r=0.8). The item categories were found to be valid and reliable. The pre and post-test was administered online.

### Data analysis

A descriptive analysis was used to examine all demographic characteristics, including age and class levels. Furthermore, a bivariate t-test and the Mann—Whitney test were used to investigate the differences in the anaemia prevention knowledge between the intervention and control groups. The data distribution was examined using skewness values. Furthermore, general linear model repeated ANOVA and the Friedman test were used to compare the effects of the video on the knowledge of anaemia between the two groups.

### Ethical consideration

The Ethics Committee Review Board of the Faculty of Health, Universitas Muhammadiyah Semarang (number 527/ KEPK-FKM/UNIMUS/2021 ) approved this study. All participants were informed that they were free to withdraw from this study any time without any consequences.

## Results

The average age of the intervention and control groups was 16.09 and 16.06 years, respectively. There were no significant differences in age, class category or anaemia knowledge between the groups before the intervention (P=0.05) (**Table 1**).

**Table 1:** Demographic characteristics of the participants (n=161).

Characteristics	Intervention (n=78)				Control (n=83)				χ^2^	P
	M±SD	Min-Max	n	%	M±SD	Min-Max	n	%		
Age	16.09±0.724	14-18			16.06±0.631	15-18				0.783^b^
*Class category*										
10th class			39	50.0			33	39.8	1.706	0.208^a^
11th class			39	50.0			50	60.2		
*Information about anaemia before the intervention*										
Never received information			72	92.3			70	84.3	2.454	0.145^a^
Received information			6	7.7			13	15.7		

a Fisher’s exact test

b Independent t-test

Three repeated measurements on the nine variables between the groups revealed a significant difference (P=0.000) (**Table 2**). The mean scores of anaemia examination barriers, anaemia susceptibility, self-efficacy to obtain iron tablets and self-recognition of anaemia signs and symptoms were significant (p<0.05). Meanwhile, anaemia severity, benefits of anaemia prevention, benefits of anaemia examination, signs of anaemia prevention and health motivation did not significantly differ (P>0.05). However, the intervention group demonstrated a significant increase in knowledge on all component variables (P=0.05), whereas the control group demonstrated a significant increase in the following three variables only (P=0.05): anaemia examination barriers, anaemia susceptibility and benefits of anaemia prevention.

In the intervention group, the following variables improved significantly (p<0.05) after the intervention (**Table 2**): anaemia examination barriers, anaemia susceptibility , anaemia severity, benefits of anaemia prevention, benefits of anaemia examination, self-efficacy to obtain iron tablets, self-recognition of anaemia signs and symptoms, signs of anaemia prevention and health motivation.

In the control group (**Table 2**), the following variables improved significantly (p<0.05): anaemia examination barriers, anaemia susceptibility and benefits of anaemia prevention. Meanwhile, the following variables did not significantly change (P>0.05).: anaemia severity , benefits of anaemia examination, self-efficacy to obtain iron tablets, self-recognition of anaemia signs and symptoms, signs of anaemia prevention and health motivation.

**Table 2:** Average knowledge of anaemia prevention among the intervention and control groups based on the nine Health Belief Model variables (n=161).

Variable	Measurement	Intervention (n=78) M±SD	Control (n=83) M±SD	P	P Intervention (n=78)	P Control (n=83)
Anaemia prevention knowledge	Pre	94±7-155	93±6.595	1.000^a^	0.000^c^	0.000^c^
	Post 1	99±5.948	94±6.953	0.000^a^		
	Post 2	102±4.26l	97±6.851	0.000^a^		
*PERCEIVED*						
Barriers to anaemia examination	Pre	19±2.536	15± 1.820	0.295^a^	0.0001^c^	0.0001^c^
	Post 1	19.5±1.513	19±1.913	0.028^a^		
	Post 2	20±0.418	20±2.332	0.000P		
Susceptibility to anaemia	Pre	6± 1.241	6± 1.478	0.447^a^	0.0001^c^	0.0001^c^
	Post 1	6±1.664	6±1.668	0.663^a^		
	Post 2	9±1.059	7± 1.604	0.000P		
Severity of anaemia	Pre	7± 1.276	7±1.393	0.875^a^	0.0001^c^	0.826^c^
	Post 1	7±1.292	7±1.392	0.811^a^		
	Post 2	7.5±1.139	7± 1.297	0.104^a^		
Benefits of preventing anaemia	Pre	10±0.819	10±0.987	0.275^a^	0.0001^c^	0.001^c^
	Post 1	10±0.559	10± 1.072	0.177^a^		
	Post 2	10±0.559	10±0.879	0.702^a^		
Benefits of anaemia examination	Pre	10±1.069	10± 1.177	0.449^a^	0.0001^c^	0.76l^c^
	Post 1	10±0.812	10± 1.177	0.472^a^		
	Post 2	10±0.812	10± 1.060	0.387^a^		
*SELF-EFFICACY*						
Self-efficacy to obtain iron tablets	Pre	15±2.209	15±2.10	0.886^a^	0.0001^c^	0.319^c^
	Post 1	15±1.778	15±2.099	0.122^a^		
	Post 2	15-5±1.045	15± 1.730	0.003^a^		
Self-recognition of anaemia signs and symptoms	Pre	7.9±1.689	7.8± 1.883	0.591^b^	0.0001^c^	0.4670.0001^d^
	Post 1	10± 1.412	7.8± 1.882	0.000^a^		
	Post 2	10± 1.412	7.9± 1.894	0.000P		
*CUES TO ACTION*						
Signs of anaemia prevention	Pre	13±1.634	13±1.713	0.857^a^	0.0001^c^	0.204^c^
	Post 1	14± 1.273	13±1.828	0.092^a^		
	Post 2	14± 1.273	13± 1.512	0.076^a^		
*HEALTH MOTIVATION*						
Health motivation	Pre	9± 1.343	8.37±1.176	0.636^a^	0.0001^c^	0.649^d^
	Post 1	8.80±1.057	8.37±1.176	0.015^b^		
	Post 2	9±1.057	8±1.298	0.041^b^		

a Mann-Whitney test

b Independent t-test

c Friedman test

d GLM repeated ANOVA

The video intervention had a large power size of 1.000 (**Table 3**). Further analysis of the nine HBM variables revealed large effect and power sizes for the benefits of anaemia examination (0.852 and 0.951, respectively). In contrast, anaemia susceptibility had a small effect size (0.402; power size: 1.000).

**Table 3:** Effect and power sizes of the animated educational video intervention in the intervention group (n=78)

Indicators	Wilks lambda		
	Effect size	Power size	P
Self-efficacy to obtain iron tablets	0.654	1.000	
Barriers to anaemia examination	0.620	1.000	
Susceptibility to anaemia	**0.402**	1.000	
Severity of anaemia	0.795	0.979	
Signs of anaemia prevention	0.733	0.999	0.000
Benefits of preventing anaemia	0.692	1.000	
Health motivation	0.726	1.000	
Benefits of anaemia examination	**0.852**	0.951	
Self-recognition of anaemia signs and symptoms	0.524	1.000	

## Discussion

Health education based on the HBM has been demonstrated to significantly improve individual behaviours, habits and beliefs as well as knowledge, attitudes and practices.^10,11^ Herein, an animated educational video based on the HBM was created to increase female adolescents’ awareness of anaemia prevention. Knowledge, attitudes, self-efficacy, reinforcing factors (family and academic factors) and supporting factors (access to information and educational resources) all influence anaemia prevention behaviours.^17^ In the present study, the video intervention significantly increased the knowledge of the female adolescents about anaemia prevention, as evidenced by the results of the first measurement, and their knowledge persisted for 4 weeks after the intervention. The use of animated educational videos significantly increases knowledge and reduces barriers to prevention.^14,18^

A factor that plays a critical role in the learning process is the education media. In recent studies, animated educational videos confined comprehensive, informative and creative information. They were efficient and appropriate for the clients’ situations and requirements.^19,20^ The combination of images and sound is more effective in increasing knowledge and changing client behaviours than single media such as images (leaflets, booklets or posters) or audio only (radio or lectures). This is why the predominance of images over characters makes information more easily received and understood,^21,22^ particularly information pertaining to action processes.

Animation videos is more interesting and easier to understand because of the high-quality images and sound, appealing images, setting and storylines tailored to the respondent’s condition. Video media are also more accessible, effective and efficient.^22^ Educational videos with audio-visuals yielded more significant results in terms of increasing knowledge, and the information obtained lasted a long time. 18 A three-dimensional material was used as an instructive medium.^23^ The selection of animation video types based on the preferences of participants can make the educational process more enjoyable and prevent boredom.^20,21,24^

Some of HBM variables were significantly improved. These findings indicated that the animated educational video was successful in transferring information to the female adolescents, as evidenced by an increase in the average knowledge level. Watching interesting and informative animated videos has also been previously reported to improve children’s knowledge, long-term memory, adherence to taking medications for secondary prevention and self-abilities.^24^ Another study found that if a female adolescent realised she was more susceptible to anaemia owing to her menstrual cycle, she could recognise and be aware of the signs and symptoms of anaemia.^25^ This condition necessitates increased iron intake as well as further investigation.^25^ The important consideration in increasing the knowledge of female adolescents will positively impact adolescent behaviours.

In the present study, some other HBM indicators did not change significantly. The insignificant increase in the knowledge of the anaemia severity in this study could be attributed to the fact that some female adolescents included had never experienced a severe form of the condition. Many female adolescents are generally unaware that they are anaemic because they do not understand the symptoms of anaemia in depth.^26^ Furthermore, most people interpret anaemia as severe when a person requires blood transfusions as part of their treatment or when a person exhibits symptoms of jaundice, splenomegaly, shortness of breath and pale tongue.^27^ Accordingly, when people believe that healthy behaviours will help them prevent the disease, they will be more likely to adopt them.^28^

Although some variables yielded no significant results, the video intervention had a large power size of 1.000 that means it can increase the knowledge of anaemia prevention among female adolescents. The effect size ranged from medium to high. Short of frequency and duration exposed may be a factor. The amount of additional information absorbed by the audience varied according to the duration of the video materials.^29^ Repeated education is required because it can increase knowledge or improve behaviours.^30^ Health belief does not adequately address some of the individual factors that affect health behaviours. The small sample size, lack of random selection and control of the comparison group may also limit the applicability of the study findings; when addressed, these factors might contribute to the improvement of the level of knowledge in the control group.

Because of the COVID-19 pandemic, research plans that were supposed to be conducted offline have moved online to avoid crowds and thus reduce COVID-19 transmission. We acknowledge some limitations of this study. The intervention necessitated the online as well as the adjustment of the viewing schedule to the school’s online learning schedule. Therefore, whether this increase in knowledge would be sustainable over time is unclear. The application of the study findings in clinical practice may be limited; the evaluation of knowledge is complex, and contributing factors need to be controlled. As this study only focused on the knowledge of the participants, the improved health outcome of reducing the incidence or manifestations of anaemia among women may not be achieved unless the knowledge is put into practice. Further studies that would evaluate the longterm applicability of the findings into practice are needed to assess the actual impact of the intervention.

## Conclusion

Based on the study findings, the animated educational video can be used as educational media to increase female adolescents’ knowledge of anaemia prevention during the COVID-19 pandemic. Educational programmes using video animation can be made more appealing and interesting to promote good health among female adolescents.
